# ‘*All That Glitters Is Not Gold*’: High-Resolution Crystal Structures of Ligand-Protein Complexes Need Not Always Represent Confident Binding Poses

**DOI:** 10.3390/ijms22136830

**Published:** 2021-06-25

**Authors:** Sohini Chakraborti, Kaushik Hatti, Narayanaswamy Srinivasan

**Affiliations:** 1 Molecular Biophysics Unit, Indian Institute of Science, Bengaluru 560012, Karnataka, India; sohini@iisc.ac.in; 2 Wellcome Centre for Anti-Infectives Research, Drug Discovery Unit, School of Life Sciences, University of Dundee, Dow Street, Dundee DDI 5EH, UK; khatti001@dundee.ac.uk

**Keywords:** quality assessment, ligand–protein crystal structures, resolution, electron density map, binding pose, PDB

## Abstract

Our understanding of the structure–function relationships of biomolecules and thereby applying it to drug discovery programs are substantially dependent on the availability of the structural information of ligand–protein complexes. However, the correct interpretation of the electron density of a small molecule bound to a crystal structure of a macromolecule is not trivial. Our analysis involving quality assessment of ~0.28 million small molecule–protein binding site pairs derived from crystal structures corresponding to ~66,000 PDB entries indicates that the majority (65%) of the pairs might need little (54%) or no (11%) attention. Out of the remaining 35% of pairs that need attention, 11% of the pairs (including structures with high/moderate resolution) pose serious concerns. Unfortunately, most users of crystal structures lack the training to evaluate the quality of a crystal structure against its experimental data and, in general, rely on the resolution as a ‘gold standard’ quality metric. Our work aims to sensitize the non-crystallographers that resolution, which is a global quality metric, need not be an accurate indicator of local structural quality. In this article, we demonstrate the use of several freely available tools that quantify local structural quality and are easy to use from a non-crystallographer’s perspective. We further propose a few solutions for consideration by the scientific community to promote quality research in structural biology and applied areas.

## 1. Introduction

Macromolecular X-ray crystallography (MX) has advanced greatly since the report of the first protein crystal structure of myoglobin (resolved at 6 Å) in 1958 [1]. Structure determination of macromolecules using crystallography has become a routine practice due to advancements in hardware and software. On the one hand, this advancement has tremendously improved our understanding of biomolecular structure–function relationships. On the other hand, such technological advances have enabled many untrained researchers to attempt MX. Other techniques such as nuclear magnetic resonance (NMR) [2] and, more recently, cryo-electron microscopy (Cryo-EM) [3] also contribute immensely to the biopharmaceutical research efforts. It is delightful to note that we are in an era that is witnessing one of the most exciting impacts of the advancement of structure determination techniques. In just about a year, more than 1000 structures (as of March 2021) of different proteins of SARS-CoV-2 (the causative agent of COVID-19) have been deposited in the Protein Data Bank (PDB) [4], and the number is likely to increase in the future. In accordance with the trend of the entire PDB [5], most of the SARS-CoV-2 protein structures currently available in the PDB were also obtained using MX [6]. The vast structural data openly available from the PDB provide an insight into the molecular mechanism of diseases, thereby accelerating drug discovery [7,8]. Reports show that structural data obtained from the PDB also drive research efforts in various interdisciplinary areas beyond the boundaries of classical structural biology [9].

High-quality crystal structures of ligand-bound macromolecules form a crucial starting point in drug discovery pipelines. The quality of the input structures greatly influences the outcomes of virtual screening experiments. However, what is referred to as a structure is a model which is built into the electron density map. Over- or under-interpretation of the density can lead to incorrect models built into the density. Mistakes can also happen either due to the bias introduced during the structure determination process or bias inherent in the crystallographer while interpreting the electron density. Often, deciphering the correct ligand (for example, small organic molecules, ions, and water) in the macromolecular binding site that fits the observed electron density is not a trivial task [10]. Hence, many expert crystallographers have expressed their concern over the quality of protein–small molecule crystal structures deposited in the PDB and recommend users to inspect the atomic co-ordinates against the electron density [11,12,13,14,15,16]. Examining a ligand’s goodness of fit to the observed electron density can be challenging for non-crystallographer users, who generally lack the training to critically evaluate the crystallographic data. This advocates for the implementation of quality metrics that are easy to interpret from a non-crystallographer’s perspective. The current PDB validation pipeline includes metrics such as the real space correlation coefficient (RSCC) and the real space R-factor (RSR) that indicate overall agreement between the ‘calculated’ and ‘observed’ electron density of a ligand and protein residues [17]. These measures are helpful but have their limitations [18]. In 2014, the PDB validation pipeline was integrated into the wwPDB (Worldwide PDB) OneDep deposition/biocuration/validation system for crystal structures. The OneDep system allows structure depositors to view the validation results prior to and during the deposition process. Pre-deposition validation reports help the depositors to make any necessary corrections before their PDB entries are made publicly available. However, comparative analysis of the PDB structures deposited before (2012–2013; ‘*Legacy group*’) and after (2014–2015; ‘*New group*’) the deployment of the wwPDB OneDep system indicates no marked improvement in the quality of the bound ligands [15]. The median and inter-quartile range of RSCC and RSR values of ligands show little or no changes between the *Legacy* and *New* groups.

Among the vast majority of the user community, the most widely used quality metric is ‘resolution’ [19]. Studies in the past have highlighted that global quality indicators such as resolution and R_free_ need not always be a good choice of metrics to judge the local quality (ligand and/or binding site residues) of a crystallographic model [11,15]. In the current work, our analysis on ~0.28 million binding sites (obtained from ~66,000 PDB entries) also echoes the fact that the quality of the local fit of a crystallographic model to its electron density is independent of the resolution at which the structure is determined. Our results show that more than half (38,693 out of 61,857; 62.5%) of the ligands identified to have major concerns (‘Bad’ quality) are determined at a resolution of 2.5 Å or better. These findings highlight the importance of assessing the quality of local regions of interest, thereby aiming to prevent any possible over-interpretation of the reported co-ordinates. Existing easy-to-use resources such as TWILIGHT [20], VHELIBS [21], EDIA [18], polder OMIT maps [22], ToBvalid [23], and the PDBe website [24,25] can be of great help to guide non-crystallographers in structure selection and local quality assessment. With the help of multiple case studies in this article, we demonstrate how some of these tools could be integrated into investigations. We believe our attempts would sensitize the users that resolution should not be the sole criterion in choosing a crystal structure as input. Besides the agreement of the reported model with experimental evidence, the ligand geometry and the stereochemical compatibility of the ligand pose with the surrounding environment are also important quality metrics that should be considered while evaluating a structure’s quality [26]. However, detailed discussion of these parameters is beyond the scope of our current study.

To retain the scientific spirit of this work, we preferred not to reveal the identity (PDB code, HET code, residue number, protein’s and ligand’s name) of the structures discussed in this article. Revealing the identity of the structures might distract the readers, leading to chances of humanizing the questionable structures with any researchers or their group. ‘*To err is human*’—given the complexities and challenges involved in determining protein–small molecule crystal structures, error-free outcomes cannot always be expected, especially from inexperienced crystallographers [27,28,29]. While experienced crystallographers can appreciate the presence of low structural quality in local regions of a high-resolution structure, non-crystallographer users are generally unaware of such problems. The main focus of this report is to sensitize the non-crystallographer user community that there can be local regions of unacceptable quality even in an atomic-resolution structure. Through this article, we also intend to encourage the users to adopt quality check protocols as an integral practice before starting with any structure-based investigations. We discuss several strategies that one can implement to assess local structural quality. Unfortunately, our policy of discussing the structures without revealing their identity prevented us from giving due credit (in the form of citations) to the authors of the publications associated with the respective structures. However, full details with disclosure of the structures’ identities and literature references to the associated structures discussed in the case studies (Section 2.3) were submitted to the journal for peer review only. The data presented in each table (main and supplementary), description provided in the figure legends, and all other relevant details required for verification of this work were provided to the reviewers and editors with disclosure of the identities of the structures. We also submitted the quality assessment reports of ~0.28 million pairs of protein–small molecule binding site and the corresponding PDB codes, HET codes, residue numbers, and chain identities for peer review.

## 2. Results

### 2.1. General Analyses

**Figure 1:**
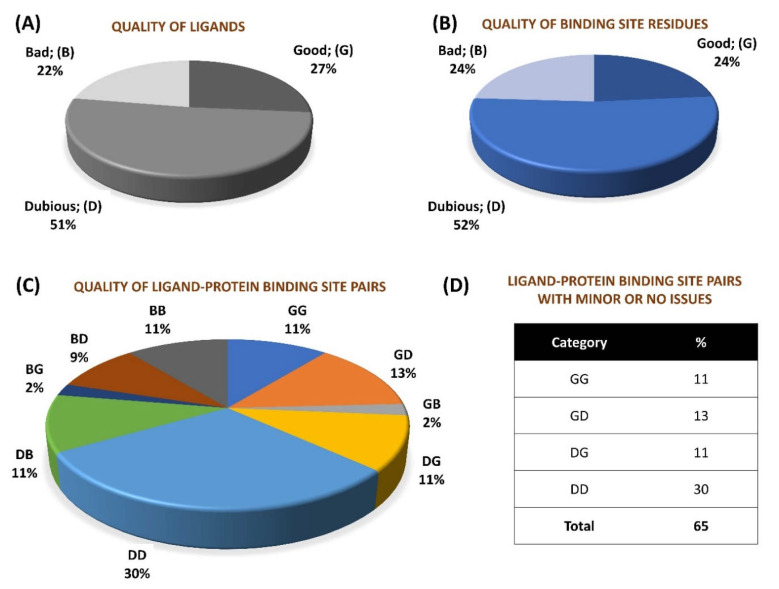
Overall percentage distribution of quality assessment results of 276,377 small molecule–protein binding site pairs in 66,851 PDB entries. (**A**) Percentage distribution of quality categories of ligands. (**B**) Percentage distribution of quality categories of protein binding sites. (**C**) Percentage distribution of quality categories of ligand–protein binding site pairs. (**D**) Chart showing percentage of ligand–protein binding site pairs devoid of ‘Bad’ quality of either ligand/protein binding site.

**Figure 2:**
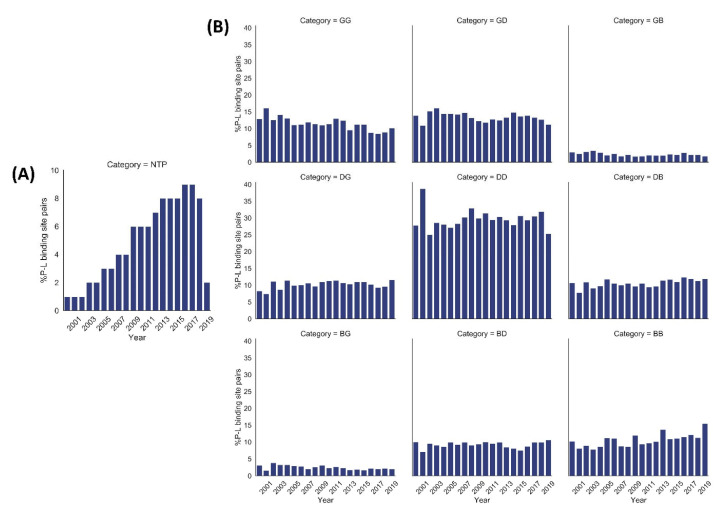
Year-wise percentage distribution of protein–ligand (P–L) binding site pairs in our dataset from 2000 to 2019 (till July). (**A**) Year-wise percentage distribution of normalized total number of pairs (NTP) of protein–ligand binding sites. The NTP values were calculated using the equation mentioned in Section 4.6. The number of protein–ligand binding site pairs shows a steady increase over the years. (**B**) Year-wise distribution of %P–L binding site pairs of each quality category based on VHELIBS assessment. The quality trend of each of the nine categories (GG, GD, GB, DG, DD, DB, BG, BD, BB) of P–L binding site pairs is almost similar over approximately the last two decades.

We performed a quality assessment of ~0.28 million pairs (derived from crystal structures corresponding to 66,851 PDB entries) of small molecule ligands and respective protein binding site residues using VHELIBS [21]. VHELIBS assigns a quality score to ligands and each binding site residue based on parameters such as RSCC, B-factors, occupancy, and a few others (for details, see Section 4.2). It then classifies the ligands/binding site residues as ‘Good’ (score = 0), ‘Dubious’ (0 < score ≤ 2), or ‘Bad’ (score > 2) based on the quality score. The VHELIBS assessment on our dataset shows that only 27% of the ligands are highly reliable, belonging to the ‘Good’ (‘G’) category. A total of 22% of the ligands are ‘Bad’ (‘B’) and need attention before using them for any practical applications, such as drug discovery/design or generating benchmark datasets to evaluate docking algorithms. A total of 51% of the remaining ligands are classified as ‘Dubious’ (‘D’) that are not highly reliable. However, these ligands do not pose a severe quality-related concern. A similar trend is observed in the quality of the binding site residues (Figure 1). In total, only a small fraction (11%) of the entire dataset could qualify, according to the eligibility criteria, for highly reliable ligand–protein binding site pairs (i.e., GG category). The inferior quality of the ligand–protein binding site pairs (i.e., BB category) comprises 11% of the whole dataset. The remaining 78% of the ligand–protein binding site pairs belong to one of the following intermediate categories: GD/GB/DG/DD/DB/BG/BD. Our analysis suggests that although the number of protein–ligand crystal structures deposited in the PDB has increased steadily over the years, the quality trend has remained more or less similar (Figure 2). For selected cases, the quality of fit of the ligands and residues was visually inspected against the corresponding electron density maps, the details of which are discussed later (Section 2.3). The complete list of the 66,851 PDB entries used for our analyses is provided as a supplementary table (Table S1).

### 2.2. Local Quality and Resolution

**Figure 3:**
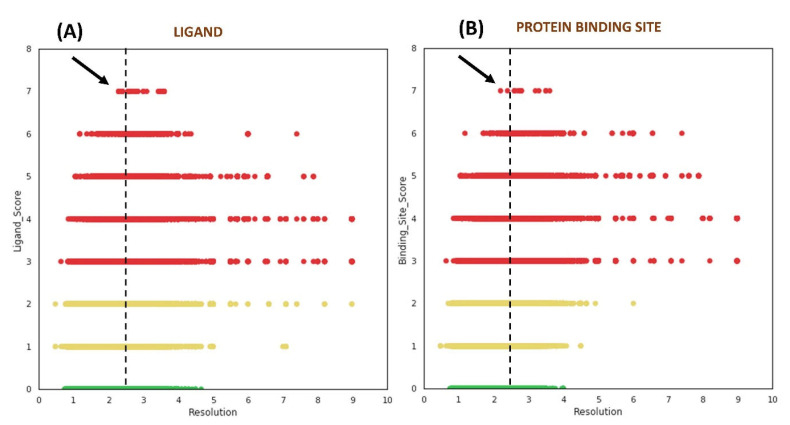
Distribution of quality scores obtained from VHELIBS vs. resolution (Å) of the corresponding structure. (**A**) Quality score of ligands vs. resolution. (**B**) Quality score of protein binding sites vs. resolution. The green, yellow, and red circles represent ‘Good’ (score = 0), ‘Dubious’ (0 < score ≤ 2), and ‘Bad’ (score > 2) categories, respectively. The vertical dashed lines in both the plots are drawn at 2.5 Å, which highlight some of the structures with the worst local quality scores (indicated by black arrows) that are solved at a resolution better than 2.5 Å. Each plot contains 276,377 data points.

We studied the distribution of VHELIBS quality scores against the resolution of the corresponding structures to understand the influence of resolution on local (ligands/protein binding sites) structural quality. This analysis revealed no clear trend between the quality of the ligands/protein binding sites and the structure’s global resolution (Figure 3). However, some apparently unexpected observations (especially from non-crystallographers’ perspective) were noted. Our analysis showed that 62.6% out of the 61,857 ligands (i.e., ~14% of the 0.28 million ligands investigated) that belong to the ‘Bad’ category are bound to crystal structures determined at 2.5 Å or better resolution. For most structure-based drug design studies, a resolution of 2.5 Å or better is generally accepted as a reliable cut-off by the scientific community and is often used as a primary filter to select structures from the PDB [30,31,32,33,34]. The analysis further indicates that it is unlikely to obtain ‘Good’ ligands in the structures with a resolution poorer than 4.5 Å. However, the ‘Bad’ ligands need not be restricted to only poorer resolution and can even be found in crystal structures determined at near-atomic resolution (better than 1.2 Å), as discussed later in case study-2 (Section 2.3.2).

### 2.3. Case Studies

In this section, we highlight some examples of resolution-independent quality concerns in ligand-bound protein crystal structures. We also demonstrate the applicability of a few freely available computational resources that can help verify the quality of the local structures. In all the subsequent sections, the proteins are denoted with the letter ‘P’ suffixed by numbers. A similar nomenclature strategy is also applied to the ligands (‘L’) and the respective complexed structure (‘C’).

#### 2.3.1. Case Study-1

This is an example of a complex, C1, comprising a protein, P1, bound to a ligand, L1. The protein in question is an important drug target for the treatment of a form of cancer.

##### VHELIBS and Visual Inspection

**Figure 4:**
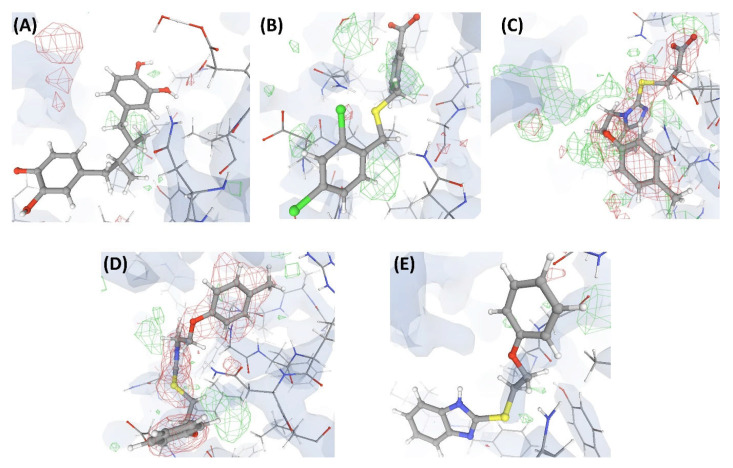
Electron density maps around the ligands bound at site S1 of the structures discussed in case study-1. (**A**) L1 in C1 (2.3 Å). (**B**) L2 in C2 (2.3 Å). (**C**) L3 in C3 (2.4 Å). (**D**) L4 in C4 (2.5 Å). (**E**) L10 in C17 (2.5 Å). The ligands are shown as a ball and stick model. The neighboring protein residues are shown as thin sticks. The blue translucent blobs are the ‘2 mFo-DFc’ maps contoured at 1.5σ, surrounding all well-determined atoms in the models. The ‘mFo-DFc’ maps (also called difference; shown as mesh) are colored as red (negative density difference contoured at -3σ) and green (positive density difference contoured at +3σ). The red density around an atom indicates either the atom is not present in the crystal or not well determined by the data or is an indicator of other aspects of incorrect modeling. The green density suggests those aspects of a structure that are reflected in the experimental data but have not been accounted for in the model. The same representation styles and contour levels are used in all the figures presented in this article. The images were generated with the 3D visualizer freely available on the PDBe website. Readers are encouraged to refer to the blog available at the PDBe website for a detailed explanation and guide [25].

In this complex, VHELIBS [21] categorized the ligand L1 as ‘Bad’ with a quality score = 7 (for details on the methodology, see Section 4.2), which is the worst score that any ligand has achieved in our dataset (Figure 3). The binding site is also categorized as ‘Bad’ (quality score = 4) and VHELIBS identified multiple residues to be examined further. Visual inspection revealed that the ligand is hardly supported by the electron density. Several binding site residues lack adequate electron density support (Figure 4). Some of these residues are mentioned in the associated publication to be crucial for interactions with the ligand L1. However, due to the inadequate support of the electron density around the residues, the reported atomic co-ordinates need attention. The associated publication reported four crystal structures including C1. These four crystal structures (C1, C2, C3, and C4) comprise an identical protein, P1—each bound to two ligands at two different binding sites (S1 and S2). The structures C1, C2, C3, and C4 are resolved at 2.3 Å, 2.3 Å, 2.4 Å, and 2.5 Å, respectively. Site S1 of P1 is occupied by four different ligands (L1, L2, L3, and L4) in each of the four crystal structures. These are the novel inhibitors identified by the authors that are expected to bind to S1, aiding in drug development against the resistant form of the cancer of interest. At S2, P1 is bound to the endogenous ligand (L’) in all four structures. Most of the currently available drugs target S2, but development of resistance is quite common. Therefore, identifying inhibitors that target a site other than S2 will aid in developing possibly better therapeutic agents against this particular form of cancer.

**Figure 5:**
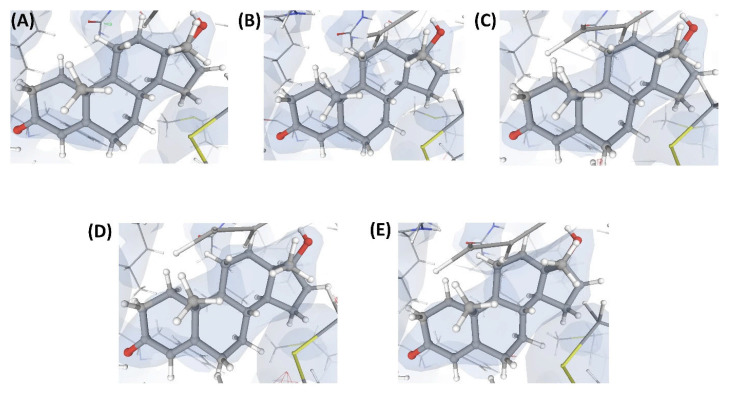
Electron density maps around the ligand L’ bound at site S2 of the structures discussed in case study-1. (**A**) C1. (**B**) C2. (**C**) C3. (**D**) C4. (**E**) C17. For details on the resolution of the structures, graphical representation styles, and color codes of density maps, kindly refer to the legend of Figure 4.

The numerical suffixes to ‘L’ indicate the corresponding complex (C1/C2/C3/C4) to which the respective ligand is bound at site S1. The ligand L’ at S2 is categorized as ‘Dubious’ by VHELIBS in all four complexes, indicating no major quality concerns. Visual inspection confirmed that L’ is adequately supported by the electron density in all the complexes (Figure 5), but the ligands bound at S1 in all four complexes are poorly supported by the electron density (Figure 4). VHELIBS analysis categorized the ligands at S1 as ‘Bad’ in three (C1, C2, and C3) out of the four structures (Table 1). In the fourth complex (C4), the ligand (L4) could not be analyzed by VHELIBS. This is because VHELIBS identified L4 to be covalently bonded to P1, and evaluation of the quality of covalently bound ligands is beyond the scope of VHELIBS. Inspection of the bound pose of L4 in P1 revealed that a few ligand atoms are involved in steric clashes with the protein binding site residues (Figure S1). The close proximity between the protein and ligand atoms possibly prompted VHELIBS to recognize L4 as a covalently bound ligand. The reported pose of L4 in C4 not only lacks electron density support but is also energetically unfavored, as evident from the steric clashes.

##### EDIA

We subjected the four complexes (C1, C2, C3, and C4) to quality evaluations using the EDIA server [18] that provides insights into the electron density support for individual atoms (for details, see Section 4.2). The EDIAm scores for the four ligands (L1, L2, L3, and L4) bound to S1 of each of the four complexes are 0.40 or below, suggesting poor atomic density support. The OPIA scores indicate only 15%, 36%, and 14% of the interconnected ligand atoms in L2, L3, and L4, respectively, are well resolved. L1 has an OPIA score of 0, indicating none of the interconnected atoms in this ligand are well resolved. The EDIA analysis suggests that L’ bound to site S2 of all the four complexes is of high/medium quality. These observations correlate well with the VHELIBS assessment report (Table 1).

One of the four ligands (L2) has a two times higher B-factor (64 Å2) than the average B-factors of the surrounding protein binding site residues (32 Å2), and all the ligand atoms are modeled with an occupancy of 0.5 (<1.0) (Table 1). Atoms of another ligand, L1, were also modeled in the respective crystal structure (C1) with an occupancy of 0.5. The B-factor of L1 (75 Å^2^) is nearly 1.4 times higher than the average B-factors of binding site residues (53.4 Å^2^). The sub-unity occupancies of the atoms in L1 and L2 indicate the presence of alternate conformations of these ligand atoms. However, the PDB files of the respective structures report only one set of co-ordinates corresponding to a single conformer of the mentioned ligands. Considering the resolution (2.3 Å) at which these structures are solved, the observations on local B-factors and occupancies of the ligand atoms are alarming [23].

In addition, the EDIA analysis indicates that the co-ordinates of some of the critical protein residues lining binding site S1 need attention (Table 1).

##### PDB-REDO

The real space correlation coefficient (RSCC) [17] of the four ligands bound to site S1 of C1, C2, C3, and C4 shows no improvement after undergoing re-refinement with the PDB-REDO pipeline [35]. The ΔRSCC for the mentioned ligands in C1, C2, C3, and C4 are −0.073, 0.027, −0.029, and 0.105, respectively.

##### Polder Maps

We assessed the polder (OMIT) maps [22] of all the ligands bound at sites S1 and S2 in the four structures using the PHENIX suite. The values of correlation coefficients (CC) calculated between three different types of maps were studied (for details, see Section 4.3). The densities in the OMIT regions of each of the four polder maps calculated for L’ molecules are likely to show the ligand atoms. However, the densities in the OMIT regions of the polder maps calculated for the S1 ligands (L1, L2, L3, and L4) are more likely to indicate bulk-solvent or noise instead of the ligand atoms. The values of CC(1,3) for the ligands bound at S1 are 0.74 or below. The CC(1,2) and CC(2,3) for these ligands are 0.65 or below. These results suggest that the weak densities of the ligands bound at site S1 are not due to bulk-solvent masking and support the concerns of low confidence of the reported ligand poses (Table S2).

The consensus results from all the above assessments indicate that the atomic co-ordinates of the ligands L1, L2, L3, and L4 in the respective PDB entries poorly fit to the electron density. However, the atomic co-ordinates of the ligand L’ bound to the same protein structures at a different site (S2) have a good fit to the electron density. Such stark differences in the quality of local regions (ligand bound to site S1 vs. site S2) within the same crystal structure were further pronounced in the results of the ligand building test and docking simulation, as discussed below.

##### Ligand Building (with ARP/wARP)

The blind fitting attempts (for details, see Section 4.4) for L1 and L2 led to the fitting of these ligands at site S2 (instead of the expected site S1 as reported in the crystal structures), with an unusually high normalized bound-state energy. Studies have shown that the normalized bound-state energy of ligands should be within −1.0 and 0.0 kcal·mol^−1^·atom^−1^ (typically −0.37 kcal·mol^−1^·atom^−1^) [36]. However, fitting of L1 and L2 at S2 yielded normalized bound-state energies of ~ 7 kcal mol^−1^ per atom and ~ 12 kcal mol^−1^ per atom, respectively. For L3 and L4, the blind fitting jobs could not be completed successfully, indicating no suitable site was found for ligand fitting. The guided fitting attempts at site S1 for L1, L2, L3, and L4 also did not yield energetically favorable ligand poses. However, the blind fitting attempts for the ligand L’ in all four structures led to fitting the ligand at site S2 with a highly favorable normalized bound-state energy (~−0.5 kcal mol^−1^ per atom). These observations raise concerns on the confidence of the reported co-ordinates of L1, L2, L3, and L4, thus supporting our earlier results (Table S3).

##### Docking Simulations

The authors reported in the associated publication that the novel S1 inhibitors were identified from a virtual screening experiment, and the docked poses of these ligands (L1, L2, L3, and L4) superimpose well on to their respective bound poses obtained from crystallographic experiments. This prompted us to perform re-docking simulations of L1, L2, L3, and L4 at site S1 and L’ at site S2. The top-ranked re-docked poses of the ligand L’ superimpose well on the reported bound pose of L’ in the respective crystal structures. However, none of the docked poses of L1, L2, L3, and L4, as predicted in our study, could reproduce the bound pose of the ligands as seen in the respective crystal structures (Figure S2).

The authors used the Glide program [37,38] for their docking studies, and we used the AutoDock vina program [39]. Both these programs are some of the top-performing docking tools and have been shown to deliver comparable results [40,41,42]. We appreciate that docking algorithms have their limitations [43,44] and fail to reproduce bound poses in many instances. However, the consistent non-reproducibility of the bound poses of the ligands (L1, L2, L3, and L4) at S1 and the consistent reproducibility of the bound poses of L’ at S2 for all four structures hint that the co-ordinates of the ligands bound at S1 might need attention.

Similar analyses, as described for case study-1, were also performed for the subsequent cases, and a similar trend in the results can be observed. We summarize only the key findings below.

#### 2.3.2. Case Study-2

**Figure 6:**
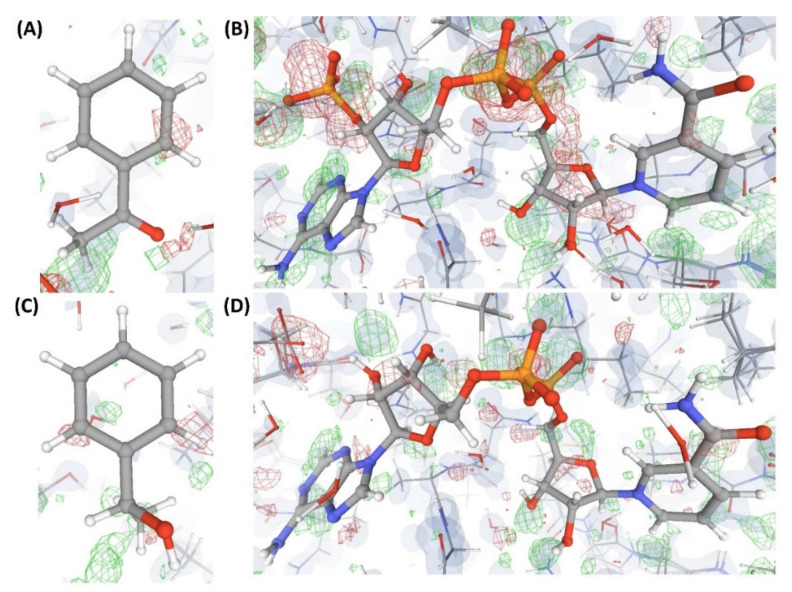
Electron density maps around the ligands (substrate and co-substrate) bound to the structures (C5 and C6) discussed in case study-2. (**A**) L” in C5 (1.0 Å). (**B**) L5 in C5 (1.0 Å). (**C**) L’” in C6 (1.1 Å). (**D**) L6 in C6 (1.1 Å). For details on graphical representation styles and color codes of density maps, kindly refer to the legend of Figure 4.

**Figure 7:**
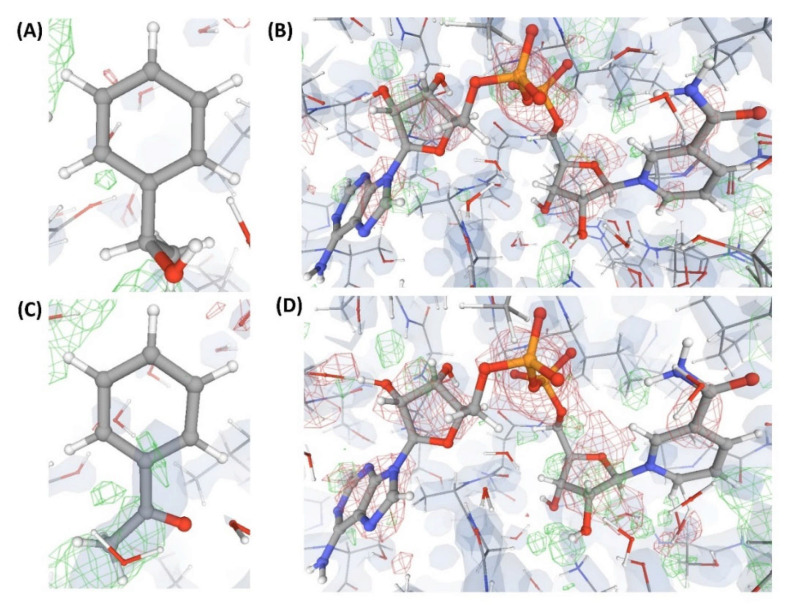
Electron density maps around the ligands (substrate and co-substrate) bound to the structures (C7 and C8) discussed in case study-2. (**A**) L’” in C7 (1.54 Å). (**B**) L7 in C7 (1.54 Å). (**C**) L” in C8 (1.78 Å). (**D**) L5 in C8 (1.78 Å). For details on graphical representation styles and color codes of density maps, kindly refer to the legend of Figure 4.

**Figure 8:**
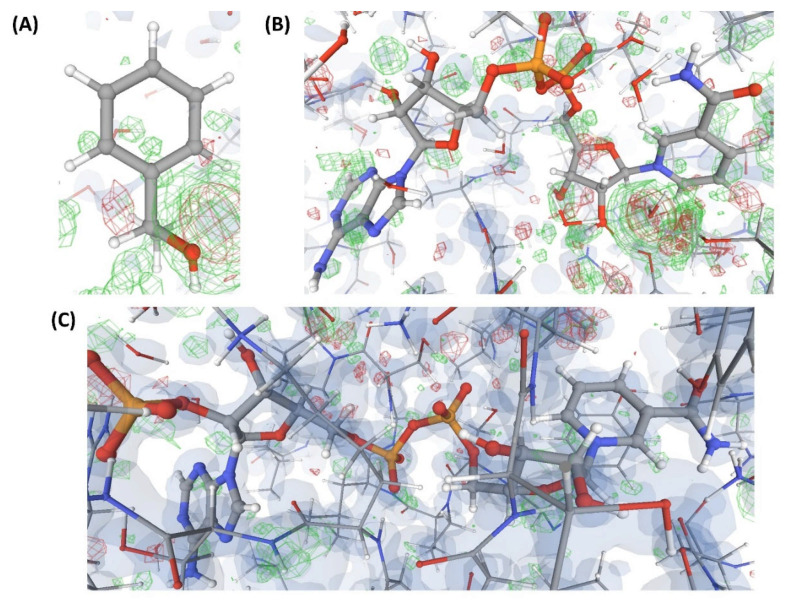
Electron density maps around the ligands (substrate and co-substrate) bound to the structures (C9 and C10) discussed in case study-2. (**A**) L’” in C9 (1.05 Å). (**B**) L7 in C9 (1.05 Å). (**C**) L5 in C10 (1.08 Å). For details on graphical representation styles and color codes of density maps, kindly refer to the legend of Figure 4.

This is an example of a crystal structure (C5) of a protein (P2) resolved at an atomic resolution (1.0 Å) that is bound to two small molecule ligands, where one is the substrate (L”), and the other is the co-substrate (L5). VHELIBS classified both these ligands and their corresponding binding sites as ‘Bad’ with a quality score = 4 for each entity. Visual inspection shows that the ligands are barely supported by electron densities (Figure 6), which is evident from their EDIA (0.10 and 0.08 for L” and L5, respectively) and OPIA scores (= 0, for both the ligands). The RSCCs (0.62 and 0.32 for L” and L5, respectively) are also poor, with no satisfactory improvement upon re-refinement by the PDB-REDO protocol (Table 2). The densities in the OMIT regions of the polder maps obtained for both the ligands are more likely to show bulk-solvent or noise instead of the ligand atoms (Table S2). The literature survey revealed that a mutant of P2 crystallized with a combination of different substrates (L” and L’”) and co-substrates (L6, L7) is deposited in the PDB as four separate entries (C6, C7, C8, and C9). All these structures (C6–C9) are resolved at high resolution (better than 1.8 Å). Visual inspection shows that all the mentioned ligands and their corresponding protein binding sites in these structures also lack fair support of electron densities (Figure 6, Figure 7 and Figure 8). Both the substrates and co-substrates have exceptionally high B-factors (>1.5 times) compared to the surrounding protein binding site residues in C5, C6, C7, C8, and C9. The poor fit of the atomic co-ordinates of some of these ligands to the electron density has also been noted in earlier published reports by other researchers. Our dataset contains another crystal structure (C10) of P2 bound to L5, which is resolved at 1.08 Å, reported by a different group of authors. VHELIBS classified L5 in C10 as ‘Good’, and visual inspection confirmed that it has an excellent fit to the electron density (Figure 8). The results of the quality assessment of all the ligands in the six structures (C5–C10) are presented in Table 2 and Table S2.

#### 2.3.3. Case Study-3

**Figure 9:**
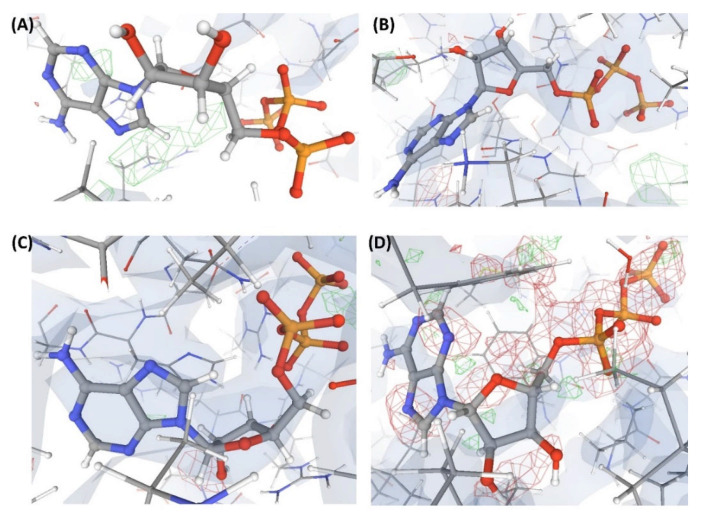
Electron density maps around the ligands bound to a few structures discussed in case study-3. (**A**) L8 in C11 (3.4 Å). (**B**) L8 in C13 (3.4 Å). (**C**) L8 in C14 (3.4 Å). (**D**) L8 in C16 (2.1 Å). For details on graphical representation styles and color codes of density maps, kindly refer to the legend of Figure 4.

This is an example of a crystal structure (C11) resolved at a resolution (3.4 Å) poorer than that of the structures discussed earlier. The authors claim to have captured a unique transient position of an endogenous ligand (L8) that binds to an enzyme (P3). VHELIBS classified L8 and its binding site as ‘Bad’. The EDIA scores (EDIAm = 0.23; OPIA = 6) and visual inspection confirmed that L8 is not supported by the electron density (Figure 9). All the atoms of L8 in C11 have an occupancy below 1. However, the alternate conformation(s) is (are) not reported. The densities in the OMIT regions of the polder map calculated for L8 are more likely to show bulk-solvent or noise instead of the ligand atoms. As 3.4 Å is a comparatively poor resolution, we explored the quality of the identical ligand (L8) or its analogues (L9) that are bound to protein structures in any PDB entries of similar resolution. Table 3 lists a few such ligands (L8 and L9) bound to P4, P5, P6, and P7 in the structures: C12, C13, C14, and C15. These ligands in C12–C15 have a fair electron density support (Figure 9). Another transient position of L8 at the same enzyme’s binding site (P3) was also reported by the same research group (that reported the structure C11) in the same year but in a different journal. This structure, C16, is resolved at a better resolution (2.4 Å) than C11. However, the atomic co-ordinates of L8 in C16 also lack electron density support (Figure 9). The densities in the OMIT region of the polder map calculated for L8 bound to C16 are more likely to show bulk-solvent or noise instead of the ligand atoms (Table S2). The VHELIBS and EDIA assessment results also agree with these observations (Table 3 and Table S2). While the re-refined structure of C11 in PDB-REDO shows no improvement in RSCC of L8, the same for C16 shows a significant (but not considerable) improvement (Table 3).

### 2.4. In Silico Assessment of Stereochemical Features of P–L Complexes

We assessed the stereochemical complementarity between selected ligands of interest and their respective binding sites as per the methodology detailed in Section 4.7. Table S4 summarizes the findings from these analyses. The normalized bound-state energy (Norm. energy) of two (L8 bound to C16 and C11) out of the three ‘Bad’ rated ligands that were analyzed is greater than 0, indicating unfavorable interactions. The interface of L8 and its binding site residues in C16 has several steric clashes. The third ‘Bad’ ligand’s (L1 in C1) Norm. energy is −0.11 kcal. mol^−1^ per atom. However, the Norm. electrostatic and Norm. van der Waals energies are 0. The local packing densities of ligands L1 in C1 and L8 in C16 are 0.49 each. The Norm. energy of the four ‘Good’ rated ligands (L9 in C12 and C15; L8 in C13 and C14) that we analyzed is less than 0. The interfaces of the ‘Good’ ligands and their binding sites are free of steric clashes. The packing densities of L9 in C12 and C15 are 0.56 and 0.64, respectively. The packing densities of L8 in C13 and C14 are 0.61 and 0.60, respectively. Few selected structures from case study-2 were subjected to steric clash and packing density analyses. The ‘Good’ ligand L5 in C10 has a packing density of 0.72, with no steric clashes. Two out of the other eight ‘Bad’ rated ligands (L5 in C8 and L7 in C7) are involved in steric clashes with their respective binding site residues. The packing densities of the other ‘Bad’ ligands, L5 in C5, L” in C5, L’” in C6, L6 in C6, L’” in C7, and L7 in C9, are 0.63, 0.47, 0.52, 0.60, 0.50, and 0.59, respectively. The ligand L4 in C4 could not be analyzed by VHELIBS, as discussed in Section 2.3.1. Our analysis shows that four atoms of ligand L4 are involved in ‘Bad’ clashes with two protein (P1) binding site residues (Figure S1). 

## 3. Discussion

Our results on the assessment of protein–ligand crystal structures show that the majority (~65%) of the structures have reasonable quality ligand and binding site residues (Figure 1). Hence, these crystal complexes may (~54% of the structures with quality categories: GD/DG/DD) or may not (~11% of the structures that are highly reliable: GG) need any attention before using them as inputs for any structure-based studies. A total of 35% of structures have either a ‘Bad’ ligand or ‘Bad’ protein binding site, or both. Notably, the extreme combinations of quality categories, i.e., GB and BG, have comparatively lower instances (~2% each), implying that it is less likely to observe a ‘Bad’ ligand in a ‘Good’ binding site and vice versa. In other words, the poor quality of one of the two binding partners (ligand or protein binding site) is likely to affect the other partner’s quality adversely. For example, the chances of seeing a ‘Good’ electron density support for a ligand in a highly flexible (manifested as a high B-factor) protein binding site that gives an ambiguous electron density (‘Bad’) are low. The fit of the model to density is influenced by the occupancy and B-factor of the atoms. A high B-factor and low occupancy would decrease scattering contributions, leading to no or poor electron density. Non-crystallographers are often unaware that atomic co-ordinates supported by no or weak electron density result from the crystallographer’s subjective interpretations. Such interpretations are not an outcome of the actual X-ray crystallographic experiments. The presence of an inadequate electron density to support the existence of a ligand could also result from bias during protein structure determination. Being unaware of such nuances may lead to over-interpretation. In the year 2008, Wlodawer et al. [45] reviewed the technical aspects of crystallography for non-crystallographers. The said article aimed to educate the users on the parameters that should be evaluated before using the structures, thereby guiding the users to obtain the best (but not more) from the published atomic co-ordinates. Our analyses in the current work agree with Wlodawer et al.’s suggestions. The general analysis presented in this report alerts the user community that ‘high-resolution’ crystal structures deposited in the PDB cannot always be relied upon without verification. With the help of the specific case studies, we also demonstrated the application of various freely available tools that aid in the inspection of local structural quality. The consensus among the results obtained from VHELIBS, the EDIA server, polder maps, and visual inspection potentiates the fact that the cases highlighted in this article need serious attention. The additional analyses involving ligand building and docking simulation, as performed for case study-1 (Section 2.3.1), are also in agreement with the aforementioned results. Our analyses on the stereochemical features of selected structures are in general agreement with the quality assessment outcomes from the crystallography data-dependent analyses (Section 2.4). It is expected that a ‘Good’ ligand is likely to obey the known stereochemical rules and exhibit favorable interactions with its surrounding environment [13]. Such expectations are independent of the structure determination techniques. Therefore, local stereochemical analyses can also be adopted for evaluating the quality of protein–ligand complexes determined by any experimental technique (crystallography/NMR/cryo-EM) or predicted by computational methods. Developing a technique-independent pipeline to assess the local quality of protein–ligand complexes is currently underway in our group. The findings from the study will be published elsewhere. Our preliminary results from the current analyses suggest that the ligands rated as ‘Good’ by VHELIBS based on crystallographic quality indicators have a better stereochemical agreement with the protein binding sites than the ‘Bad’ ligands. Unlike the ‘Good’ ligands, the ‘Bad’ ligands are generally less favorably accommodated, less densely packed, and engaged in unjustified steric clashes.

Through the analyses reported in this article, we aim to contribute to improving the quality control practices in structural biology research in the ways discussed below.

### 3.1. Sensitizing Users

Many users of crystal structures most often rely on the global quality indicator ‘resolution’ to select input structures from the PDB for virtual screenings or other computational structure-based studies. As it was discussed earlier (Section 2.2), it is common practice to discard poorer-resolution structures by defining 2.5 Å as a resolution cut-off. Our analyses suggest that the ‘Bad’ ligand/protein residues are not restricted to structures of poor resolution (Figure 3). Therefore, users should carefully choose the input structure as its quality has a crucial impact on the experiment’s outcomes. In general, a high-resolution protein–ligand crystal structure has lesser uncertainty in the position of atomic co-ordinates than its low-resolution counterparts. However, the global ‘resolution’ of the structure need not necessarily guarantee that the reported position and conformation (pose) of ligand/binding site residues (local regions) agree with the experimental observations. The fit of the model to the electron density map is a primary indicator of the local model quality and is quantified by the metric RSCC. Nevertheless, in some instances, the quality is not evident from the RSCC alone [18] (Table 1).

We acknowledge that visual inspection of electron density maps (Fo-Fc and 2Fo-Fc) for the local model is the best approach to infer the goodness of fit of the model to experimental observations. However, visual inspections might not be easily comprehended by novice users. If the density arising from a local region is weak, then the flat bulk-solvent model (which is most often used by the present-day crystallographic refinement programs) may further obscure the density. Decision making for such cases that involve weak densities in the regions of interest can be challenging. We recommend using multiple freely available tools that provide a quantitative understanding (such as VHELIBS, EDIA, and polder maps) to verify the reliability of atomic co-ordinates in the regions of weak densities. Recently, Yao et al. developed a new metric that can help evaluate structural data with a better physicochemical context [46]. Users may also consider exploring this new metric that derives units of electrons directly from electron density maps to indicate the local structural quality. This tool is available as a Python package (pdb-eda).

If an improved re-refined model of a protein–ligand complex is available in the PDB-REDO database, it may be preferred over the PDB model for any practical applications [47]. We encourage users to explore the possibilities of optimizing the protein–ligand complexes deposited in the PDB using combined quantum mechanics/molecular mechanics (QM/M) approaches [48]. Studies have shown that QM/MM optimization of the originally deposited models in the PDB often improves the ligand and binding site geometry. Improvement in the ligand geometry may also reduce ligand strain and help improve the interaction profile between the bound ligand and the binding site residues [49,50,51]. In many instances, the co-ordinates of the QM/MM-optimized models are found to have better agreement with crystallographic quality metrics, such as the standard R, R_free_, and real space R values. It has also been shown that non-covalent interactions at the protein–ligand interfaces are generally underestimated in structures determined at a resolution poorer than 1.5Å [52]. However, the underestimated interactions can be recovered after QM/MM optimization. Thus, QM/MM optimization of available crystal complexes can provide better starting points for structure-based drug discovery programs [53].

### 3.2. Re-Visiting the Structures of Concern by Crystallographers

The authors of the structures with quality concerns might consider re-visiting the respective projects and providing explanations on the quality of their structures and its implications. They may also re-visit the PDB entry of their structure and resubmit a better built model. Attempts to improve the quality of the deposited ‘Bad’ structures either by re-refinement or re-determination would be a last resort. This can ensure that the broader user community, which is not trained to analyze the complex crystallographic data, can focus better on effectively utilizing the data for further research without worrying about their quality.

### 3.3. Proposals to the Journals

An analysis by Brown and Ramaswamy in 2007 [54] indicated that crystal structures of proteins published in prestigious journals tend to have more errors. One of the reasons the authors pointed out is the involvement of higher complexities and broader requirements for accepting an article by the premier journals (*“the results should be novel and important for science as a whole, not solely crystallography”*) [54]. We also found that some of the structures with quality-related concerns at the protein-ligand interfaces, as discussed in this article, were published in reputed journals. While investigating the first case (as detailed in Section 2.3.1), we noted a figure of electron density maps around the ligands as provided by the authors in the associated article. The figure clearly shows that the ligands (L1, L2, L3, and L4) have no or feeble density support. It could be possible that the said figure remained unnoticed during the peer review, and eventually the article was published. The biochemical assays reported in the associated publication show that the ligands L1, L2, L3, and L4 bind P1 with micromolar affinities (IC50 ranges from ~13 to 50 μM), which are supposedly not strong binders. The authors designed a series of lead molecules based on the structure–activity relationship studies of these ligands. One of these molecules (L10) with better activity (IC50 = 4.2 μM) than the previously reported hits was crystallized with P1, and results were published in the same journal in the following year. In the L10-P1 complex (C17; 2.5 Å), while L10 is bound at site the S1 of P1, the site S2 is occupied by L’. The quality assessment details of these ligands are provided in Table 1 and S2. In agreement with the quality of the earlier structures, the ligand L10 at S1 of C17 has poor electron density support, but the atomic co-ordinates of L’ at S2 have adequate support of the experimental electron density (Figure 4 and Figure 5). In-depth probing of the associated literature revealed that while the authors participated in the molecular modeling, medicinal chemistry, and biochemical experiments, all the crystallographic experiments reported in both the publications were performed by an external organization as a part of contract research. It could be possible that the authors were not intensely involved in analyzing the crystallographic data and their quality. Given the broad scope of the concerned journal, the possibility that the manuscripts were reviewed by biologists/chemists who are less experienced in critically examining crystallographic data cannot be ruled out. To effectively handle such situations, we propose that the journals and various bodies of the International Union of Crystallography may consider implementing systems that mandate submissions of easily interpretable quality reports of local structural data in the form of EDIA and polder map assessment reports. This can help non-crystallographer stakeholders quickly access the quality reports for decision making. Towards the goal of having a full-proof quality check in place, we encourage computational chemists, biologists, and other users of crystal structures to include analyses of quality scores (EDIA, correlation coefficients from polder maps for regions of interest with weak density) of the input structures in their publications. Journals may consider implementing the requirement for inclusion of such data in computational and related studies. This can prevent the propagation of misleading results due to possible faulty input structures. We have already embraced the practice of including discussions on quality assessments of input structures in our publications that can be referred to in [55,56,57].

### 3.4. Proposals to the Repository Community

The structure repository community (PDB) is urged to adopt stringent quality assessment policies, especially for protein–small molecule complexes. Various policies that include archiving the raw data and implementation of correctional measures upon detecting an error have already been suggested by many experts [58,59]. Initiatives to establish an independent international committee to periodically monitor the quality of the PDB structures have also been taken [60,61]. Additionally, from a non-crystallographer’s perspective, we propose that the PDB validation report should integrate metrics that provide users with a better understanding of the local quality by quantifying how well each atom (especially for the ligands of interest and their binding sites) is supported by the electron density. The RSCC, as available from the PDB validation reports, serves as a local quality indicator. However, unlike EDIA scores, it does not provide atomic-level understandings. As discussed earlier, verifying the existence of atoms in regions of weak densities is challenging if the densities are masked by a bulk-solvent density. Under such circumstances, polder map assessment can aid decision making. Therefore, integrating EDIA and polder map assessment into the existing PDB validation pipeline could be helpful to users. For the already deposited structures, EDIA and polder map assessment on ligands and protein binding site residues should be performed. The results from these assessments should be made publicly available to educate the users on the quality of the deposited structures. Explanations from the authors should be mandated where the parameters deviate from the standard criteria. Policies should be in place to correct any detected error within a stipulated time period with documentation of the reasons for removal of faulty structures. We believe the suggested efforts can complement the existing quality control measures and help maintaining the integrity of the structural repositories. In the words of Minor et al., these measures can help in “*safeguarding structural data repositories against bad apples*” [59].

## 4. Materials and Methods

We assessed the quality of protein–small molecule crystal complexes deposited in the Protein Data Bank (PDB) [4] against the available crystallography-dependent parameters. Selected cases were subjected to detailed studies. The tools and/or resources and the methodology adopted for the analysis are elaborated below.

### 4.1. Dataset

All small molecule (70 Da ≤ molecular weight ≤ 800 Da) bound protein structures were downloaded from the PDB in July 2019. Only those structures where the small molecule contains at least one carbon atom were considered for further analysis. This initial dataset comprised ~92,000 unique PDB entries.

### 4.2. Quality Assessment of Protein–Ligand Binding Sites

Henceforth, only the structures determined using X-ray crystallography were considered for quality assessment. The quality of the protein–ligand binding sites in all the crystal complexes was assessed using the tool Validation HElper for LIgands and Binding Sites (VHELIBS) [21]. Given a protein–ligand (non-covalently bound) crystal complex, this tool categorizes the binding site of the input structure in either of the following nine possible classes: (i) Good/Good (GG), (ii) Good/Dubious (GD), (iii) Good/Bad (GB), (iv) Dubious/Good (DG), (v) Dubious/Dubious (DD), (vi) Dubious/Bad (DB), (vii) Bad/Good (BG), (viii) Bad/Dubious (BD), and (ix) Bad/Bad (BB). The first quality label indicates the bound ligand’s quality, and the second label indicates the quality of the protein binding site. Hence, if a protein–ligand binding site is labeled as ‘Good/Good’ or ‘GG’, it indicates that the ligand and all the protein binding site residues have a quality score = 0, suggesting highly reliable atomic co-ordinates. For VHELIBS assessment, we defined the binding site as a region that consists of residues within 4.5Å of the bound ligand. We preferred to use the abbreviated forms of the quality labels, denoting each category with its first letter for the ease of our discussion. VHELIBS assessed the bound ligand and each protein binding site residue of the input structures against the parameters mentioned below and their corresponding specified limits:

(a) Maximum Real Space R-factor (RSR): 0.4;
(b) Maximum ‘Good’ RSR: 0.24;
(c) Minimum ‘Good’ real space correlation coefficient (RSCC): 0.9;
(d) Average occupancy: 1.0;
(e) Occupancy weighted average B-factor (OWAB): 50 Å^2^;
(f) Maximum R_free_: 0.3;
(g) Maximum [(R_free_) − (R_work_)] value: 0.05.

Deviation from the specified limits for any of the above parameters would lead to a penalty with an increment in the quality score of ‘1’. Therefore, if there is no deviation from the specified limits of all the mentioned parameters, the score assigned to the ligand/binding site residue is 0. The entity (ligand/binding site residue) is then categorized as ‘Good’ (G). If there are deviations with respect to, at most, any two parameters (0 < score ≤ 2), then the entity is labeled as ‘Dubious’ (D). A ligand/protein residue that is labeled as ‘Dubious’ may or may not be of severe concern. Such a structure may be re-classified by the user as ‘Good’ or ‘Bad’ after visual inspection, depending upon the criticality of the question that the user aims to address using the input structure. A score > 2 categorizes the particular entity as ‘Bad’ (B) and serious attention before using the structure for any practical applications might be needed. A higher score indicates a poorer quality of the ligand/residue. The protein binding site is assigned a quality label based on the highest score that any residue receives among all the residues within the bound ligand’s specified radius. Thus, even if a single residue in a protein binding site receives a score >2, the binding site will be labeled as ‘Bad’ by VHELIBS. The program lists such residues for examination. Hence, it is advisable that the user should visually examine the ligand and residues while performing analysis with a small dataset to decide on further applicability of the structure. The default ligand ‘exclusion’ list of VHELIBS was modified so that all ligands (except water or similar solvents and metal ions), irrespective of their biological relevance, are considered for the analyses. The crystal structures deposited in the PDB without the information on respective structure factor files could not be assessed. A total of 66,851 unique PDB entries corresponding to ~0.28 million (276,377) pairs of non-covalently bound small molecule ligands and protein binding site residues could be evaluated.

For selected cases, detailed quality assessments were performed using the EDIA tool [18] available at the Proteins*Plus* webserver (https://proteins.plus/; we last accessed this website on 19 May 2021) [62]. The electron density score for individual atoms (EDIA) quantifies the electron density fit of an atom. The EDIA tool combines the atomic EDIA values with the help of the power mean to compute EDIAm, the electron density score for any entity (small molecules, fragments, or residues). EDIA score ≥ 0.8 indicates satisfactory electron density support for the entity. A score of 0.4 ≤ EDIA score < 0.8 suggests medium electron density support for the atoms, and a score below 0.4 means poorly supported atoms. An EDIAm below 0.8 indicates at least three atoms in the ligand/residue under consideration have an EDIA below 0.8. Additionally, the tool provides another score called OPIA, overall percentage of well-resolved interconnected atoms. This score allows distinguishing between overall poorly supported ligands and ligands with partially unsupported substructures. The PDB-REDO database containing re-refined structures of many existing PDB entries was also consulted for selected example cases to check improvements in RSCC [35].

### 4.3. Polder Map Assessment

The tool *polder.phenix* implemented in the *PHENIX* software suite was used to generate polder (OMIT) maps to test the quality of ligands [22]. An OMIT map is computed by excluding the selected atoms from the structure and updating the corresponding structure factors, followed by calculating a map. It is expected that the electron density for the omitted (excluded) atoms will be seen as positive features in the OMIT map if the atoms are actually present in the crystal structure. Polder (OMIT) maps do not apply bulk-solvent flattening around the selected OMIT regions (which are the ligand atoms in our study) to better assess the presence of ligands (the electron density of which might be obscured sometimes due to bulk-solvent flattening). The polder approach computes three maps: m1, m2, and m3. Maps m1 and m2 are calculated using synthetic data (*F_obs_* = *|F_model_|*), one assuming that the omitted atoms are present (m1) and the other assuming that the omitted atoms are absent (m2). Map m3 is the polder map that uses actual experimental data. Local correlation coefficients (CC) between all pairs of the three maps are then computed. By design of the test, m1 is expected to show omitted atoms, and m2 is expected to show a bulk-solvent density. If the polder map (m3) shows the omitted atoms, it is expected to best correlate with m1. On the contrary, if m3 shows bulk-solvent, it is expected to correlate best with map m2. However, if the omitted atoms are highly mobile (as indicated, for example, by high B-factors) and/or the resolution of the structure is low, m1 may instead be smeared. It could then resemble the bulk-solvent map, generating a high correlation with map m2. Therefore, a sufficiently higher CC between m1 and m3 than the other two pairs of CC (m1–m2 and m2–m3) suggests that the polder map is likely to show the omitted atoms. The inputs for generating a polder map for selected omit regions include two files: the model (.pdb) and reflection data files (.mtz) as available from the PDB.

### 4.4. Ligand Building

The ARP/wARP program webservice was accessed through https://arpwarp.embl-hamburg.de/ (we last accessed this website on 19 May 2021) to build the ligands in the density maps of the selected structures [36] by using: (a) respective phase information and (b) the atomic co-ordinates of the protein, available in the PDB. The ligand fitting trials were performed in the following ways: (a) blind fitting, and (b) guided fitting. For the blind fitting, the program was instructed to place the ligand at the best site of the input protein structures. For the guided fitting, co-ordinates of approximately the central atom of the ligands (as available from their corresponding PDB files) were chosen, and the ARP/wARP program was instructed to search for a suitable ligand position within a 3 Å radius of the specified set of co-ordinates. The program also reports normalized energy for the ligands fitted in the respective protein binding site, which serves as a validation parameter to indicate the fit’s goodness in terms of favorable interactions with the protein binding site. Normalized bound-state energy should be within −1.0 and 0.0 kcal.mol^−1^.atom^−1^ (typically −0.37 kcal.mol^−1^.atom^−1^) [36]. A value above 0.0 kcal.mol^−1^ per atom indicates a likely case of ligand misfit, which is not energetically favored by the protein binding site.

### 4.5. Docking Simulation

Guided docking simulations within pre-defined grid boxes (that were centered around the ligands of interest) were performed for case study-1 using AutoDock vina [39] to generate 20 binding poses per ligand with an energy range of 9 kcal mol^−1^.

### 4.6. Visualization and Graph Plotting

The academic version of PyMol (Schrödinger, LLC) was used for visualizing the molecules. The Mol* 3D viewer on the PDBe website was used to visualize the electron density maps around the ligands and generate the images [24,25]. Microsoft Excel and Python seaborn libraries [63] were used for plotting the graphs. In Figure 2, the year-wise percentage of protein–ligand (P–L) binding site pairs in each category is calculated as below.

(1) $$\%P-L\;\mathrm{binding}\;\mathrm{site}\;\mathrm{pairs}=\frac{\mathrm{Number}\;\mathrm{of}\;P-L\;\mathrm{binding}\;\mathrm{site}\;\mathrm{pairs}\;\mathrm{in}\;\mathrm{the}\;\mathrm{particular}\;\mathrm{category}\;\mathrm{in}\;a\;\mathrm{given}\;\mathrm{year}\times 100}{\mathrm{Total}\;\mathrm{number}\;\mathrm{of}\;P-L\;\mathrm{binding}\;\mathrm{site}\;\mathrm{pairs}\;\mathrm{in}\;\mathrm{the}\;\mathrm{dataset}}$$

### 4.7. In Silico Approach to Assess Stereochemical Quality of P–L Binding Sites

For selected cases, the quality of P–L binding sites was assessed against the following stereochemical features: (i) steric clashes, (ii) energy, and (iii) ligand packing density. The P–L pairs with missing residues/atoms (within 6 Å of the bound ligand) in the electron density maps of the respective binding sites could not be considered for the analyses. Only the cases with extreme ligand quality ratings (‘Good’ and ‘Bad’) as assigned by VHELIBS were included in the analyses. The calculations of the mentioned stereochemical parameters are based on the reported 3D co-ordinates of the ligand/residues. Therefore, the results are influenced by the inherent co-ordinate errors of the structures. Generally, the poorer the resolution of the structure, the larger the co-ordinate errors. Hence, the values of different parameters reported here for each structure are limited by their resolution.

The steric clashes at the interfaces of the proteins and small molecules of interest were analyzed using the CLASHSCORE module [64] in the PHENIX software suite. This module calculates all-atom contacts and classifies an overlap of greater than or equal to 0.4 Å as a ‘Bad clash’. Only the non-hydrogen atoms of the ligand and the protein binding site residues that are not engaged in hydrogen bonding are considered for reporting in our steric clash analyses.

The bound-state energy of the ligand pose reported in the respective PDB files was calculated using the AutoDock scoring function implemented in the compute_AutoDock41_score.py script available in the MGLTools package. The input ‘pdbqt’ files of the proteins and the ligands required for the energy calculations were prepared using the prepare_receptor4.py and prepare_ligand4.py scripts from the MGLTools package available for proteins and small molecules, respectively [65]. The Autodock4 scoring function is based on a semi-empirical energy force field. The overall energy score comprises the following components: electrostatics, hydrogen bonding, van der Waals, torsion, and desolvation [66]. The energy scores of each P–L pair were normalized against the total number of non-hydrogen atoms in the respective ligands to avoid the influence of the ligand size on energy calculations. An energy score above 0 indicates an unfavorable interaction between the protein and the ligand. A more negative score indicates better stereochemical complementarity between the two binding partners. The energy calculations in this study did not consider any water/ion/other ligand-mediated interactions between the protein binding site and ligand of interest. Therefore, we did not perform energy calculations on the structures that contain more than one ligand of interest in the protein binding sites to avoid possible under-estimation of energy scores. These are the structures discussed in case-study 2, where the enzyme active sites are occupied by two ligands—a substrate and a cofactor, each influencing the interaction profile of the other.

The local packing densities of the ligand atoms were calculated using the Voronoia program [67]. The packing density of atoms involved in steric clashes are unreliable and are therefore not considered for reporting. The packing density values range from 0 to 1. The higher the value, the denser the atomic packing.

### 4.8. Software Details

The following versions of the different software were used in this study.

(a) VHELIBS version 4.3 [68]
(b) Autodock Vina version 1.1.2 [69]
(c) Polder map module available in Phenix 1.19.2-4158 [70,71]
(d) Clashscore module available in Phenix 1.19.2-4158 [70,71]
(e) PyMol version 2.4.0 [72]
(f) Voronoia version 1.0 [73]
(g) MGL Tools version 1.5.6 [74]

## 5. Conclusions

The structural co-ordinates submitted in the PDB serve as the important starting points of many interdisciplinary research projects in both academia and industries. In particular, the drug discovery programs are heavily dependent on the availability of high-quality structural data of protein–ligand complexes. Our analyses of ~0.28 million binding sites derived from crystal structures corresponding to ~66,000 PDB entries revealed that only 11% of the small molecule and protein binding site pairs are highly reliable. In contrast, the remaining 89% pairs might need little or serious attention before usage. Previously, many expert crystallographers have suggested users to verify the quality of the structural data by inspecting the experimental data. However, most users of crystal structures are non-crystallographers who are not trained to comprehend the complex crystallographic data. Hence, such analyses can be time-consuming and challenging to perform, especially for large-scale data investigations in bioinformatics-centric studies. To tackle this problem, we encourage users to explore the application of more straightforward tools that quantify the goodness of fit of individual atoms (EDIA) and, in case of weak densities, verify the presence of the atoms of interest by masking the bulk-solvent density (polder maps). Through the various case studies discussed in this article, we sensitize the user community about the old saying ‘*all that glitters is not gold*’. The first case study (Section 2.3.1) demonstrates how the confidence on the atomic co-ordinates of two ligands bound to different sites within the same protein can starkly vary even when the structures are resolved at a modest resolution (~2.5 Å or better). The second case study highlights structures with near-atomic resolution (Section 2.3.2), but the confidence associated with the reported ligand co-ordinates is low. The third case study (Section 2.3.3) shows an example of a crystal structure with a comparatively lower resolution (3.4 Å) and a poor support of the atomic co-ordinates by electron density. Our analyses show that co-ordinates of many other similar ligands (identical/chemical analogues) crystallized with other proteins and resolved at the same resolution (3.4 Å) have moderately better agreement with their corresponding electron density maps. Therefore, the users must be vigilant about the local regions’ quality, irrespective of the resolution at which the crystal structure is determined. Further, our preliminary findings on exploring stereochemical features as local quality indicators of protein–ligand complexes agree with the evaluation reports based on crystallography-dependent data.

Having sensitized the users about the prevailing problems, we also proposed a few solutions for the scientific community at large to consider for discussion and implementation. We note that some of our proposals resonate with the initiatives and recommendations discussed by Minor et al. [59]. Archiving raw experimental data in publicly available repositories would immensely help verify the quality of the local regions in a model. However, the usage of such data is likely to be restricted to only expert users with sound knowledge of crystallography. Hence, we believe complementing the data archival initiatives by integrating metrics that express local models’ quality in a quantifiable manner would help non-crystallographers quickly verify the structural data quality. In the years to come, these initiatives would boost quality research practices in the broad area of structural biology. Finally, we would like to state that ‘*absence of evidence is not evidence of absence*’. Hence, the lack of electron density support for a region of interest does not necessarily mean that the respective atoms were absent during the experiment. There can be several reasons, as discussed earlier, that can lead to no or poorly interpretable electron density. Users should be careful about such structures and must apply their general physical chemistry understandings to judge if a local region that does not pass the quality checks could be used as an input for their research to obtain meaningful biological insights.

## Figures and Tables

**Table 1:** Quality assessment of protein–ligand binding sites in C1–C4, and C17 (case study-1).

Entity (Ligand/Residue)	VHELIBS *					EDIA Analysis																				PDB Validation Report				
	Ligand Score; Binding Site Score; Category					EDIAm ^a^					OPIA ^b^					Mean B-Factor ^c^					Occ. < 1.0 ^d^					RSCC ^e^				
	C1	C2	C3	C4 ^$^	C17	C1	C2	C3	C4	C17	C1	C2	C3	C4	C17	C1	C2	C3	C4	C17	C1	C2	C3	C4	C17	C1	C2	C3	C4	C17
Ligand at S1	**7;4;** **BB**	**6**;2; **B**D	**4;3;** **BB**	NA	**5;3;** **BB**	**0.22**	**0.38**	**0.39**	**0.27**	**0.41**	**0**	**15**	**36**	**14**	**0**	75.01 (53.41)	**64.07** (32.05)	19.96 (35.96)	19.97 (39.87)	**82.52** (41.12)	**22**	**20**	0	0	**19**	**0.61**	**0.23**	**0.67**	**0.69**	**0.78**
S1: Phe1	NA					**0.56**	0.90	0.81	**0.69**	**0.70**	**27**	91	73	**36**	**36**	56.53	29.01	35.90	45.28	38.92	0	0	0	0	0	0.93	0.93	0.95	0.94	0.95
S1: Asn55	NA					**0.74**	**0.67**	**0.70**	**0.67**	**0.54**	75	50	75	**63**	**38**	59.71	41.85	38.72	46.98	53.29	0	0	0	0	0	**0.87**	**0.87**	**0.86**	**0.79**	0.94
S1: Phe154	NA					**0.79**	**0.77**	**0.73**	0.80	**0.68**	73	55	55	**45**	**45**	57.93	37.09	43.20	45.71	55.62	0	0	0	0	0	0.90	0.93	0.93	0.93	0.94
S1: Glu157	NA					**0.36**	**0.35**	**0.70**	**0.55**	**0.76**	**44**	56	56	**44**	**44**	54.83	32.04	36.58	38.39	35.86	**3**	**3**	**3**	**3**	0	0.96	0.96	0.94	0.93	0.98
S1: Leu158	NA					**0.63**	0.90	**0.76**	0.82	**0.79**	75	75	75	75	75	51.19	29.96	31.86	35.88	29.28	0	0	0	0	0	0.96	0.98	0.98	0.96	0.93
S1: Glu165	NA					0.87	0.84	0.91	0.91	0.84	56	56	78	89	67	57.10	36.72	41.59	47.95	38.21	0	0	0	0	0	0.91	0.95	0.96	0.96	0.92
S1: Arg168	NA					**0.38**	**0.33**	**0.33**	**0.26**	**0.59**	**45**	**36**	**36**	**36**	**45**	60.94	45.93	45.71	56.23	62.37	**6**	**6**	**6**	**6**	**1**	0.96	0.94	0.94	0.92	0.93
Ligand at S2	2;**3**; D**B**	1;1; DD	1;1; DD	1;2; DD	1;1; DD	0.88	0.93	0.87	0.86	0.85	71	90	71	71	67	39.96 (44.30)	23.01 (25.99)	24.32 (29.96)	24.14 (34.64)	25.24 (29.73)	0	0	0	0	0	0.94	0.95	0.98	0.97	0.97

The het codes of the ligands at S1 of C1 (2.3 Å), C2 (2.3 Å), C3 (2.4 Å), C4 (2.5 Å), and C17 (2.5 Å) are L1, L2, L3, L4, and L10, respectively. The het code of the ligands at S2 in all the five structures is L’. The important residues at site S1 that lack fair electron density support are listed in the table. Kindly note the residue numbers as given in the PDB are not revealed in accordance with our principle of masking the identities of the structures due to the reason stated in the text. The numbers used here are with reference to the first residue (Phe) in the table which is assigned as residue number ‘1’ in this paper. * A ligand/binding site is classified as ‘Bad’ (B) by VHELIBS when the score is above 2, indicated in bold. ^$^ As explained in the text, the quality assessment of the ligand (L4) bound at site S1 of C4 could not be performed with VHELIBS. ^a^ An EDIAm score of any fragment (ligand/residue) below 0.8 indicates at least three atoms in that fragment are not well supported by electron density. The values below 0.8 are shown in bold. ^b^ OPIA: overall percentage of well-resolved interconnected atoms; the values below 50 (shown in bold) indicate less than 50% of the interconnected atoms in the particular fragment have good electron density support. ^c^ B-factors are measured in units of Å^2^. The numbers within the brackets, ‘( )’, in the first and last row indicate the average B-factors of the binding site residues around the respective ligand. These values were calculated by averaging the mean B-factor of the protein residues (that are within 4.5 Å from the ligand) obtained from the EDIA server. Wherever a ligand’s B-factor is 1.5 times more than that of its surrounding protein residues, the B-factor of the former is shown in bold, and it demands careful inspection. ^d^ Occ. < 1.0: number of atoms in the fragment that have an occupancy less than unity; cases where one or more atoms have Occ. < 1.0 are shown in bold. Notably, the ligands bound at S1 of C1, C2, and C17 have 22, 20, and 19 non-hydrogen atoms, respectively. None of the atoms in these ligands have Occ. = 1.0. ^e^ RSCC: a score below 0.9 indicates the atoms in the ligand/residue are not well supported by electron density and is shown here in bold. The scores are taken from the respective PDB validation report.

**Table 2:** Quality assessment of ligands bound to the structures C5–C10 (case study-2).

Protein–Ligand Complex Identifier (Resolution)	VHELIBS *		EDIA Analysis								PDB Validation Report		PDB-REDO	
	Ligand Score; Binding Site Score; Category		EDIAm ^a^		OPIA ^b^		Mean B-Factor ^c^		Occ. < 1.0 ^d^		RSCC ^e^		ΔRSCC ^f^	
	SL	CSL	SL	CSL	SL	CSL	SL	CSL	SL	CSL	SL	CSL	SL	CSL
C5 (1.00 Å)	**4; 4; BB**	**4; 4; BB**	**0.10**	**0.08**	**0**	**0**	**63.29** (13.80)	**94.82** (14.32)	0	0	**0.62**	**0.32**	0.281 (significant)	0.226 (significant)
C6 (1.10 Å)	**4; 3; BB**	**3; 4; BB**	**0.07**	**0.11**	**0**	**0**	**55.43** (9.72)	**63.60** (10.85)	0	0	**0.10**	**0.16**	0.188 (significant)	0.279 (significant)
C7 (1.54 Å)	**4; 4; BB**	**4; 4; BB**	**0.24**	**0.18**	**0**	**0**	**71.94** (15.65)	**52.55** (16.45)	0	0	**0.14**	**0.34**	**−0.016** **(insignificant)**	**−0.017** **(insignificant)**
C8 (1.78 Å)	2; **3**; D**B**	**3**; 2; **B**D	**0.51**	**0.18**	**33**	**0**	**49.47** (14.40)	**49.78** (14.42)	0	0	**0.62**	**0.32**	**−0.034** **(insignificant)**	**−0.024** **(insignificant)**
C9 (1.05 Å)	2; **3**; D**B**	**3**; 2; **B**D	**0.04**	**0.1**	**0**	**0**	**55.04** (13.63)	**67.08** (14.27)	0	0	**0.37**	**0.12**	0.153 (significant)	0.240 (significant)
C10 (1.08 Å)	N/A	0; 0; GG	N/A	0.84	N/A	73	N/A	5.5 (6.25)	N/A	0	N/A	N/A	N/A	0.004 (significant)

The het codes of the substrate ligands (SL) in C5, C6, C7, C8, and C9 are L”, L’”, L’”, L”, and L’”, respectively. The het codes of the co-substrate ligands (CSL) in C5, C6, C7, C8, C9, and C10 are L5, L6, L7, L5, L7, and L5, respectively. *A ligand/binding site is classified as ‘Bad’ (B) by VHELIBS when the score is above 2, indicated here in bold. ^a^ An EDIAm score of any fragment (ligand/residue) below 0.8 indicates at least three atoms in that fragment are not well supported by electron density. The values below 0.8 are shown in bold. ^b^ OPIA: overall percentage of well-resolved interconnected atoms; a value below 50 (shown in bold) indicates less than 50% of the interconnected atoms in the particular fragment lack good electron density support. Notably, most of the ligands (except the substrate in C8 and co-substrate in C10) have an OPIA score = 0. ^c^ B-factors are measured in units of Å^2^. The numbers within the brackets, ‘( )’, indicate the average B-factors of the binding site residues around the respective ligand. These values were calculated by averaging the mean B-factor of the protein residues (that are within 4.5 Å from the ligand) obtained from the EDIA server. Wherever a ligand’s B-factor is 1.5 times more than that of its surrounding protein residues, the B-factor of the former is shown in bold, and it demands careful inspection. ^d^ Occ. < 1.0: number of atoms in the fragment that have an occupancy less than unity. ^e^ RSCC: a score below 0.9 indicates the atoms in the ligand/residue are not well supported by electron density and is shown here in bold. The scores are taken from the respective PDB validation report (if available). ^f^ ΔRSCC: change in RSCC after re-refinement. A negative value indicates worse in PDB-REDO. The values in bold indicate insignificant change between final and initial density map fits (quantified by RSCC). Although significant changes are observed for the ligands in C5, C6, and C9, the density fits are not satisfactory to give a high RSCC. N/A: not applicable.

**Table 3:** Quality assessment of ligands bound to the structures C11–C16 (case study-3).

Protein–Ligand Complex Identifier; Ligand Code (Resolution)	VHELIBS *	EDIA Analysis				PDB Validation Report	PDB-REDO
	Ligand Score; Binding Site Score; Category	EDIAm ^a^	OPIA ^b^	Mean B-factor ^c^	Occ. < 1.0 ^d^	RSCC ^e^	ΔRSCC ^f^
C11; L8 (3.4 Å)	**7**; **6**; **BB**	**0.23**	**6**	79.20 (69.83)	**31**	**0.58**	**0.080** **(insignificant)**
C12; L9 (3.4 Å)	0; **3**; G**B**	0.88	81	106.33 (111.43)	0	0.93	**0.070** **(insignificant)**
C13; L8 (3.4 Å)	0; **3**; **BB**	**0.74**	61	109.38 (98.70)	0	0.95	0.058 (significant)
C14; L8 (3.4 Å)	0; 2; GD	0.84	77	47.55 (44.04)	0	0.95	−0.180 (significant)
C15; L9 (3.4 Å)	0; 1; GD	**0.72**	56	49.71 (49.17)	0	0.97	−0.055 (significant)
C16; L8 (2.1 Å)	**4**; 1; **B**D	**0.31**	**19**	**61.20** (24.36)	0	**0.59**	0.190 (significant)

* A ligand/binding site is classified as ‘Bad’ (B) by VHELIBS when the score is above 2, indicated here in bold. ^a^ An EDIAm score of any fragment (ligand/residue) below 0.8 indicates at least three atoms in that fragment are not well supported by electron density. The values below 0.8 are shown in bold. ^b^ OPIA: overall percentage of well-resolved interconnected atoms; a value below 50 (shown in bold) indicates less than 50% of the interconnected atoms in the particular fragment are well resolved. ^c^ B-factors are measured in units of Å^2^. The numbers within the brackets, ‘( )’, indicate the average B-factors of the binding site residues around the respective ligand. These values were calculated by averaging the mean B-factor of the protein residues (that are within 4.5 Å from the ligand) obtained from the EDIA server. Wherever a ligand’s B-factor is 1.5 times more than that of its surrounding protein residues, the B-factor of the former is shown in bold, and it demands careful inspection. ^d^ Occ. < 1.0: number of atoms in the fragment that have an occupancy less than unity. Notably, L8 has 31 non-hydrogen atoms, and all these 31 atoms of L8 in C11 have occupancy less than 1.0. ^e^ RSCC: a score below 0.9 indicates the atoms in the ligand/residue are not well supported by electron density and is shown here in bold. The scores are taken from the respective PDB validation report. ^f^ ΔRSCC: change in RSCC after re-refinement. A negative value indicates worse in PDB-REDO. The values in bold indicate insignificant change between final and initial density map fits (quantified by RSCC).

## Data Availability

Not applicable.

## Supplementary Materials

The following are available online at https://www.mdpi.com/article/10.3390/ijms22136830/s1.
