# Supplementary material for: ‘All That Glitters Is Not Gold’: High-Resolution Crystal Structures of Ligand-Protein Complexes Need Not Always Represent Confident Binding Poses
Source: Int J Mol Sci. 2021 Jun 25;22(13):6830. doi: 10.3390/ijms22136830 (PMC8268033; doi:10.3390/ijms22136830)
Supplement: Supplementary file 1 [file ijms-22-06830-s001.zip › Supplementary_Figures.pdf]

'All that glitters is not gold': High-resolution crystal structures of ligand-protein complexes need not always represent confident binding poses.

Sohini Chakraborti[1], Kaushik Hatti[2], Narayanaswamy Srinivasan[1*]

[1]Molecular Biophysics Unit, Indian Institute of Science, Bengaluru, Karnataka 560012, India
[2]Drug Discovery Unit, Wellcome Centre for Anti-Infectives Research, School of Life Sciences, University of Dundee, Dow Street, Dundee. DDI 5EH, U.K
[*]Correspondence: ns@iisc.ac.in; Telephone: +91-80-22932837; Fax: +91-80-23600535.

Supplementary Figures:

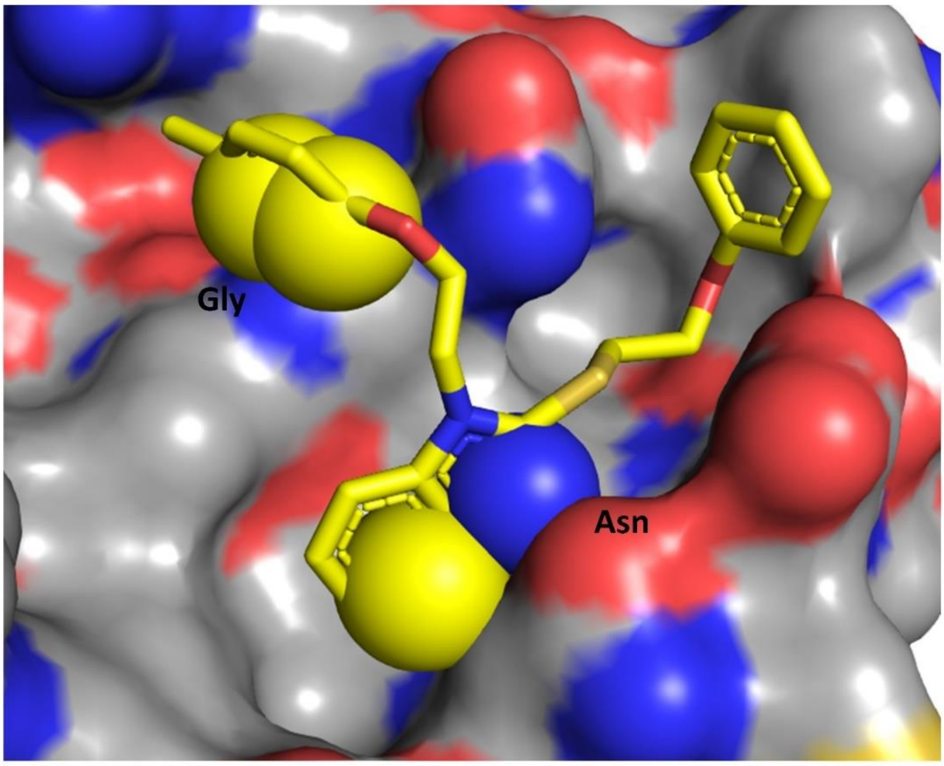

Figure S1: **Interatomic 'Bad' clashes between four ligand atoms of L4 and two protein residues (Gly and Asn)**. Five steric clashes between four ligand atoms and two protein residues are observed with atomic overlaps: 0.696, 0.704, 0.772, 0.604, and 0.585.

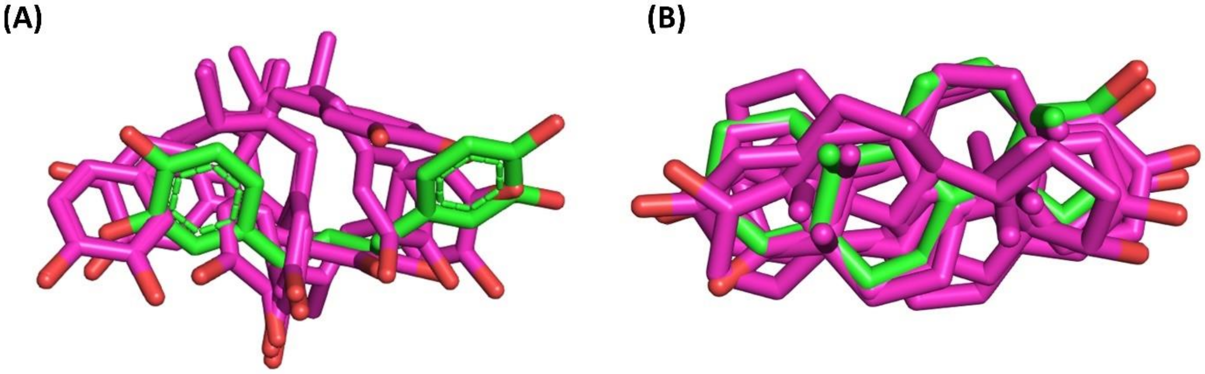

Figure S2: Superposition of re-docked (magenta) poses of ligands on their respective bound pose (green) reported in C1. (A) L1 and (B) L'. This is a representative example. Similar results have been observed for the other structures discussed in case study-1 (section 2.3.1).
